# 'Score to Door Time', a benchmarking tool for rapid response systems: a pilot multi-centre service evaluation

**DOI:** 10.1186/cc10329

**Published:** 2011-07-27

**Authors:** Kieran J Oglesby, Lesley Durham, John Welch, Christian P Subbe

**Affiliations:** 1 Department of Anaesthesia and Critical Care, Weston General Hospital, Grange Road, Uphill, Somerset, BS23 4TQ England, UK; 2 North of England Critical Care Network: Outreach Group, North Tyneside General Hospital, North Shields, Rake Lane, Tyne and Wear, NE29 8NH England, UK; 3 Critical Care Unit, University College Hospital, Euston Road, London, NW1 2BU England, UK; 4 Departments of Acute Medicine and Intensive Care, Wrexham Maelor Hospital, Croesnewydd Road, Wrexham, LL13 7TD Wales, UK

## Abstract

**Introduction:**

Rapid Response Systems were created to minimise delays in recognition and treatment of deteriorating patients on general wards. Physiological 'track and trigger' systems are used to alert a team with critical care skills to stabilise patients and expedite admission to intensive care units. No benchmarking tool exists to facilitate comparison for quality assurance. This study was designed to create and test a tool to analyse the efficiency of intensive care admission processes.

**Methods:**

We conducted a pilot multicentre service evaluation of patients admitted to 17 intensive care units from the United Kingdom, Ireland, Denmark, United States of America and Australia. Physiological abnormalities were recorded via a standardised track and trigger score (VitalPAC™ Early Warning Score). The period between the time of initial physiological abnormality (Score) and admission to intensive care (Door) was recorded as 'Score to Door Time'. Participants subsequently suggested causes for admission delays.

**Results:**

Score to Door Time for 177 admissions was a median of 4:10 hours (interquartile range (IQR) 1:49 to 9:10). Time from physiological trigger to activation of a Rapid Response System was a median 0:47 hours (IQR 0:00 to 2:15). Time from call-out to intensive care admission was a median of 2:45 hours (IQR 1:19 to 6:32). A total of 127 (71%) admissions were deemed to have been delayed. Stepwise linear regression analysis yielded three significant predictors of longer Score to Door Time: being treated in a British centre, higher Acute Physiology and Chronic Health Evaluation (APACHE) II score and increasing age. Binary regression analysis demonstrated a significant association (*P *< 0.045) of APACHE II scores >20 with Score to Door Times greater than the median 4:10 hours.

**Conclusions:**

Score to Door Time seemed to be largely independent of illness severity and, when combined with qualitative feedback from centres, suggests that admission delays could be due to organisational issues, rather than patient factors. Score to Door Time could act as a suitable benchmarking tool for Rapid Response Systems and helps to delineate avoidable organisational delays in the care of patients at risk of catastrophic deterioration.

## Introduction

Cardiopulmonary arrests and emergency admissions to Intensive Care Units (ICUs) from general wards are often preceded by a prolonged, detectable period of physiological deterioration [1,2]. Mortality increases with the extent of physiological derangement, as indicated by vital signs, prior to ICU admission [3,4]. Delays in both the initial recognition of the deteriorating patient, as well as the prompt initiation of treatment, are a further, significant contributor to morbidity and mortality of emergency admissions to ICUs [5].

Heterogeneous Rapid Response Systems (RRS), including Medical Emergency Teams (MET), Rapid Response Teams (RRT) and Critical Care Outreach (CCO), have evolved to satisfy local requirements of healthcare systems. Having initially developed in Australia, RRS are now commonplace in US and UK centres, supported by statutory bodies, such as the Royal College of Physicians [6] and the National Institute for Health and Clinical Excellence [7]. Their common goal is to identify and intervene as early as possible in the deteriorating patient to avoid further, preventable critical events and so reduce inhospital morbidity and mortality.

However, the principles of RRS have been the subject of ongoing debate [8], while the negative outcome of the only multi-centre study has been attributed to the complexity of the intervention. There have also been documented problems in the intervention arm with delayed responses to deteriorating patients [9]. Literature reviews have recommended that large prospective randomised controlled trials are needed to confirm the efficacy of these interventions [10-12].

Historically, quality assurance systems in healthcare have been weak and this has led to a significant gap between recommended practice and 'on the ground' reality [13]. Evolving inhospital practices should be analysed in a structured and critical manner to provide objective evidence of performance and effectiveness. In this context, there is a lack of benchmarking tools for RRS to facilitate comparison between centres and processes, and to promote regular internal quality assurance beyond the frequency of cardiopulmonary arrests [14]. This is likely to significantly hamper service evaluation and interventional trials [15]. Given the title of the intervention, it would appear reasonable to assume that benchmarking tools should record whether individual RRS are rapid and responsive.

In analogy to the 'Door-to-Needle' time for thrombolysis of ST-elevation myocardial infarctions [16], we propose the development of a benchmarking tool - 'Score to Door Time' (STDT) - for the process of emergency admissions to ICU via RRS. Such a tool would enable individual centres to identify the features causing delays in emergency inpatient transfers, and monitor and improve internal efficiency of individual RRS. Ultimately, this may help address systemic delays in the care process which contribute to the morbidity and mortality of critically ill patients.

Accordingly, the aims of this service evaluation were to 1) review timing of ICU admission processes for patients identified by RRS, 2) design a simple benchmarking tool for this process, 3) establish whether the tool can be successfully applied to different RRS approaches, 4) determine the factors which can predict variation in STDT, and 5) suggest a reference point for the timeliness of RRS.

## Materials and methods

This pilot service evaluation was designed as a pragmatic multi-centre collaboration. Formal ethical approval for the study was not required by NHS Medical Research Information Service (MRIS) criteria [17]. Collaborators in the contributing international centres obtained permission to perform data collection as per local protocols, typically via registration with clinical audit departments. Consequently, patient consent was not deemed necessary as these were individual audits of consultants' patients. Prospective data collection was performed in 17 European, North American and Australian hospitals in total (see acknowledgements) during the period from August 2009 to January 2010. Collaborators working within various RRS were approached using a standardised letter outlining the aims and objectives of the evaluation, proposed method of data collection and subsequent analysis. Only emergency admissions to ICUs from within the admitting hospital were eligible for inclusion. Participating centres were requested to review data from a minimum of five emergency admissions to ICU. Information was obtained from entries in existing patient case records, including medical and nursing notes. All patient data were anonymised at the point of collection. Collaborators were subsequently requested to state whether there was a delay in transfer to ICU and the potential reasons.

**Figure 1:**
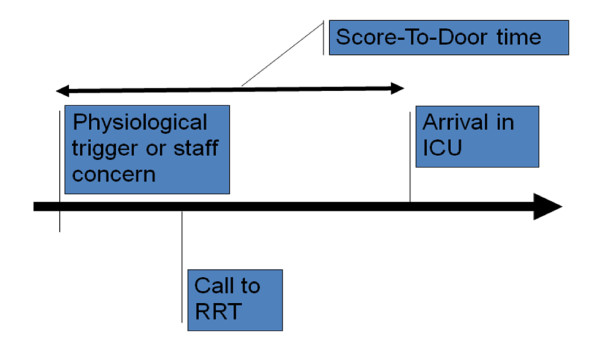
**Patient journey from general ward to ICU**. RRT, rapid response team.

The trigger time was defined as the time of the first recorded physiological abnormality that should have triggered a call-out of the local RRS or, alternatively, the actual time of call-out if not related to a physiological abnormality (for example, nursing staff concerned about a patient). The trigger had to be followed by a persistent physiological abnormality until the time of ICU admission. Bedside observations at the trigger time were obtained from medical records and included: blood pressure, pulse rate, respiratory rate, oxygen saturations, fraction of inspired oxygen, temperature, level of consciousness and urine output. In cases where the RRS was not appropriately triggered when a significant physiological abnormality was first documented, the time delay for the call-out of the RRT, MET or CCO was also recorded (see Figure 1: Patient journey from general ward to ICU).

Illness severity of patients was stratified using a validated scoring model demonstrating high sensitivity and specificity for hospital mortality within 24 hours - the VitalPAC™ Early Warning Score (ViEWS) (The Learning Clinic, Pynes Hill, Exeter, Devon, England) [18]. ViEWS scores were calculated retrospectively from the bedside physiological observations provided by individual centres, in order to facilitate fair comparison. No centre was employing ViEWS at the time of data collection. Severity of illness on admission to ICU was stratified with the Acute Physiology and Chronic Health Evaluation Scores (APACHE II) [19]. STDT was defined as the period between trigger time and time of admission to ICU, stated to be the time of first physiological observations in ICU. Data were processed using SPSS™ (version 18) (International Business Machines Corp, Armonk, New York, USA). Linear regression was performed to assess the impact of case mix factors, including age, sex, illness severity, centre location, speciality and time of deterioration on STDT.

## Results

### Patient and centre characteristics

We received data regarding 177 patients. The majority of contributors (13 of 17) were from the UK, submitting 80.2% (142) of total datasets. Centres submitted between 3 and 35 patients (mean 10 patients, standard deviation 5). Of the hospitals, nine were university teaching hospitals and eight were district general hospitals. The mean number of inpatient beds per centre at the time of data collection was 611 (range 188 to 1,010) and number of ICU beds 20 (range 5 to 60). A total of 109 patients were male and 58 were female, with 10 datasets (Centre B) not disclosing patient gender. Ninety-seven patients were admitted from medical wards and 46 from surgical wards. Alternative sources of admissions included the emergency room (n = 12) and other hospital departments. Ten of the 177 triggers were cardiac arrest calls (5.5%). Table 1 summarises epidemiological and physiological data from the patients at the time of RRS trigger.

**Table 1:** Bedside observations at time of Rapid Response System call-out with admission APACHE II score and ViEWS.

Parameter	n	Mean	Standard Deviation
Age	167	60	20
Systolic blood pressure (mmHg)	164	124	37
Heart rate	163	109	25
Respiratory rate	163	25	9
Oxygen saturations (%)	164	92	8
Inspired oxygen (%)	162	48	30
Temperature (°C)	116	37.1	1.2
ViEWS	167	8	3
APACHE II	145	18	8

APACHE, Acute Physiology and Chronic Health Evaluation; ViEWS, VitalPAC™ Early Warning Score

### 'Score to Door Time'

The time between either a physiological trigger or call-out of RRT and the subsequent ICU admission (STDT) was a median of 4:10 hours (interquartile range (IQR) 1:49 to 9:10, n = 177). Time from physiological trigger to call-out of RRT was a median 0:47 hours (IQR 0:00 to 2:15, n = 120). Time from call-out to ICU admission was a median of 2:45 hours (IQR 1:19 to 6:32, n = 120).

STDT was a median 4:32 hours (IQR 2:24 to 10:03, n = 142) for UK patients and a median of 1:41 hours (IQR 00:47 to 3:15, n = 35) for non-UK patients. The difference between the UK and non-UK centres was statistically significant and based on a difference in the time from call-out to ICU admission (*P *< 0.0001, Mann-Whitney U test) rather than a difference in the time between trigger and ICU admission (*P *= 0.336).

**Figure 2:**
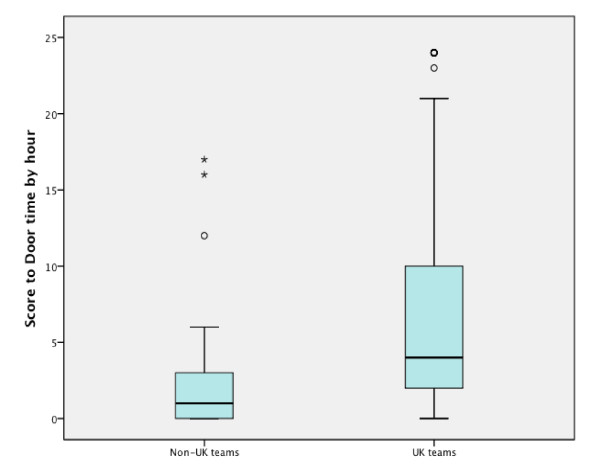
**Comparison of mean Score to Door Times for British and non-British Rapid Response Systems**.

During normal working hours (9:00 to 17:00 hours) STDT was a median of 4:00 hours (IQR 1:45 to 8:56) versus a median of 4:12 hours (IQR 2:00 to 9:22) outside normal working hours. There was considerable variability between centres, to be interpreted in the context of the small sample sizes (see Table 2 and Figure 2).

**Table 2:** Score to Door Time (STDT) for each centre

Centre	Number of patients	Median STDT (hours)	Standard deviation
A	10	04:30	06:17
B	10	01:24	00:52
C	5	01:16	02:34
D	14	05:02	07:46
E	5	04:05	06:01
F	22	08:15	26:20
G	11	06:33	04:43
H	10	01:37	01:19
J	5	02:20	02:35
K	5	09:48	20:07
L	5	01:30	08:12
M	5	04:30	20:41
N	3	01:50	08:32
O	18	04:20	16:48
P	7	03:05	08:12
Q	7	15:00	15:53
R	35	04:00	04:43
Total	177	04:10	13:31

### Admission delays

Collaborators provided qualitative descriptions of causes for unnecessary delays and increased STDT. It was suggested that there were no delays for 50 patients. Overall, causes for delay were classified as clinical (56 patients, 44.1%), non-clinical (28 patients, 22.0%) or not apparent (43 patients, 33.9%). Clinical reasons included initial improvements after treatments on the ward (n = 4), diagnostic or therapeutic procedures prior to ICU admission (n = 12), a need to stabilise the patient prior to transfer (n = 9) and an intercurrent cardiopulmonary arrest.

Of those patients with STDT of more than four hours (n = 94), there were only 14 cases (14.9%) where a clinical reason for delay could be established. Forty of those 94 (42.6%) had no discernable reason for a delay, whilst the remaining 40 (42.6%) indicated in their comments organisational problems, including waiting for senior reviews (n = 11), the lack of an available ICU bed (n = 14), and insufficient staffing.

### 'Score to Door Time' predictors

Stepwise linear regression analysis yielded three significant (*P *< 0.05) predictors of STDT when adjusted for other factors: centre location, patient age and APACHE II score. All centres, save for P and R, had at least one dataset with insufficient information to accurately calculate APACHE II scores. Patients in non-British centres had significantly shorter STDT (*P *= 0.016) than British centres. Age was a significant predictor of reducing STDT (*P *= 0.013); for every year of age STDT was reduced by 0:09 hours. Higher APACHE II scores were significantly correlated (*P *= 0.039) with longer STDT, with each point increase predicting a further 0:18 hours until ICU admission. All other factors were non-significant predictors. Table 3 summarises the effect of these factors.

**Table 3:** Stepwise linear regression with Score to Door Time as the dependent variable (adjusted R-squared = 0

Factor	Regression coefficient	95% Confidence interval	*P-v*alue
Admission 0900 to 1700	-0.059	-6.858 to +3.467	0.517
Age	-0.203	-0.269 to -0.032	0.013
APACHE II	+0.039	+0.015 to +0.573	0.039
Gender	-0.030	-6.294 to +4.495	0.742
Medicine (versus other speciality)	+0.124	-6.659 to +4.908	0.765
Non-British Centre	-0.198	- 12.93 to -1.380	0.016
ViEWS	+0.054	-0.586 to +1.065	0.566

APACHE II, Acute Physiology and Chronic Health Evaluation II score; ViEWS, VitalPAC™ Early Warning Score.

**Figure 3:**
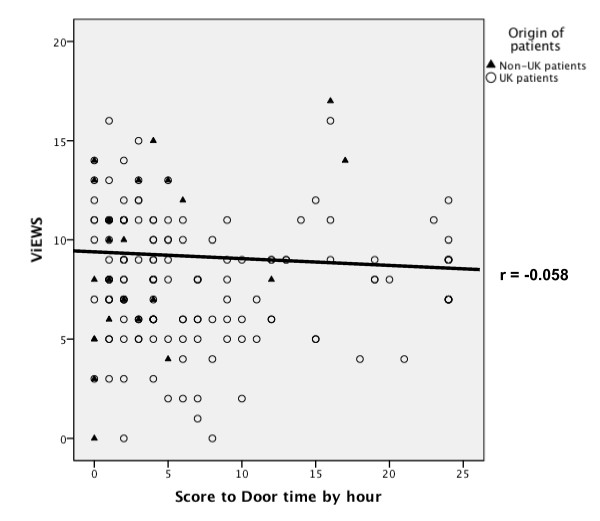
**Comparison of Score to Door Time (STDT) and VitalPAC™ Early Warning Score (ViEWS)**. Scatterplot with regression line demonstrating no significant correlation between STDT and ViEWS as a marker of illness severity.

Binary logistic regression analysis (see Table 4) demonstrated an association of APACHE II scores >20 with a STDT of more than four hours (*P *< 0.045, 0.309, 95% CI 0.31 to 1.50) in British patients. Correlation between ViEWS and STDT was weak (Spearman's r = -0.058, *P *< 0.02) (see Figure 3). These suggest that greater severity of illness at the time of RRS contact did not lead to timelier admission.

**Table 4:** Binary logistic regression for **Acute Physiology and Chronic Health Evaluation (**APACHE) II score >20

Factor	Regression coefficient	95% Confidence interval	*P-v*alue
Gender	+0.053	0.472 to 2.355	0.897
Admission 0900 to 1700	-0.385	0.309 to 1.495	0.680
STDT >4 hours	+0.871	0.309 to 1.495	0.045
ViEWS >8	+0.049	0.475 to 2.323	0.903

STDT, Score to Door Time; ViEWS, VitalPAC™ Early Warning Score

## Discussion

This 'snapshot' service evaluation collected data from a range of RRT, MET and CCO in a range of international healthcare systems. The information gathered suggests that, in many cases, RRS respond swiftly after recording of abnormal physiological triggers. However, in a significant number of cases, there are considerable delays and these are not correlated with severity of illness at the time of detectable deterioration, thus contributing to higher APACHE II scores at ICU admission. Patients with higher APACHE II scores, in addition to their acute physiological derangement, tend to be more elderly and have a greater degree of underlying comorbidity and chronic organ insufficiency. In almost one-third of non-clinical delays, attending ICU teams were waiting for senior ICU and parent team input regarding the suitability of patients for escalation of care. Of these, 78.0% had APACHE II scores greater than 20 and 84.6% were greater than 70 years of age.

In a further third of delayed admissions, no clear reason could be established by collaborators and were not explained by clinical interventions. We suggest that this is likely to be due to inherent inefficiencies of admission systems, especially when combined with the information that when collaborators did establish reasons, they represented a variety of 'organisational' reasons. Given that increasingly unwell patients (represented by higher APACHE II scores) were not admitted more swiftly to ICU, this may also indicate that current systems are not able to respond more promptly. Delays in recognition and treatment in the pre-RRS era have been shown to affect morbidity and mortality of critically ill patients on general wards [20]. The restricted availability of critical care beds [21], particularly in British centres, as well as the associated complex referral systems which limit access, appear to be factors that delay critical admissions [22,23]. While RRS were introduced to help prevent unnecessary ICU admissions [23], they cannot function in isolation.

We have measured, where possible, the time from the physiological trigger to call-out of the RRT and, subsequently, from that point to ICU admission. While the first interval depends on recognition of critical illness by staff on general wards, the second interval is largely dependent on critical care processes and resources, as well as the duration of interventions. We believe that STDT is the simplest and most unambiguous parameter to measure as it measures the performance of the whole system, as opposed to parts of the system. Clinicians in a number of settings were able to use the tool; they included UK-style CCO teams using Early Warning Scores, as well as METs and RRTs using lists of call-out criteria. We have only been able to address some factors leading to delays. This question is now part of a follow-on project using human factors analysis to quantify the avoidable and unavoidable delays, as well as their impact on care. We would suggest that a delay of more than the median time of four hours for procedures and observations might be inappropriate as this delay was associated with increased APACHE II scores on admission to intensive care.

Current benchmarking of RRS is often focussed around the number of cardiopulmonary arrests [9,14]. It has proved difficult to ascertain whether reductions in arrest numbers are due to an increase in Do Not Attempt Resuscitation orders and palliative interventions, or caused by improved outcomes due to timely treatment. Given that critical illness with organ failure is ideally treated in an ICU [24], STDT provides critical care departments with a tool to assess the functionality of a RRS independent of the trigger system used. STDT can thus serve as a starting point for process mapping of ICU admissions and identification of potentially modifiable factors which lead to delays.

In this study, we have addressed the time interval between a physiological trigger and ICU admission. It has been rightly pointed out that some RRTs deliver ICU-style interventions including invasive haemodynamic monitoring, inotropic support and invasive ventilation on general wards. A more relevant time for benchmarking may, therefore, be the time from trigger to active organ support regardless of patient location. Similarly, we recognise that it may often be entirely appropriate that STDT is prolonged, depending on local circumstances and individual patient factors. For example, prior to admission to an ICU bed, urgent imaging might establish a definitive diagnosis more efficiently and help to avoid multiple intra-hospital transfers of an unstable patient. Therefore, the use of STDT as a tool to assess the performance of a RRS is perhaps best considered as an adjunct to other outcome measures.

The data collection was part of routine clinical practice and it is possible that recording lagged behind the actual performance of transfers to ICU and that fluctuating physiological variables might impair the measurement of the trigger time. However, participants were given detailed explanations and a graphic depiction on how to determine the trigger time. Additionally, the consistency of results seems to suggest that this is probably a lesser issue. Data collection was voluntary and it is, therefore, feasible that results are not truly representative. However, if we consider that the mean STDT in the majority of British centres was greater than six hours, it suggests our findings are likely to be accurate.

The sample size of our study was too small to examine whether delays in admission to ICU of patients actively managed by RRTs affect mortality or morbidity. We believe that we have set the foundations for future examination of the impact of delays on outcomes by demonstrating the extent of variability of STDT and the association with higher APACHE II scores. Further research is needed to clarify this key issue. The ongoing Sepsis Pathophysiological and Organisational Timing (SPOT) study is likely to make a major contribution in answering this question [25].

## Conclusions

The absence of universally accepted process measures for RRS has meant that testing and improving RRS has proved difficult. 'Score to Door Time' is the first tool to enable meaningful service evaluation and benchmarking of these heterogeneous approaches. The precise impact of delays on morbidity and mortality in response to deterioration of patients on general wards remains to be quantified.

## Key messages

• There is no benchmarking tool to enable meaningful comparison of heterogeneous Rapid Response Systems in the escalation of care of critically ill patients.

• A novel tool was tested in this international service evaluation of 17 centres, demonstrating a median Score to Door Time of 4:10 hours (IQR 1:49 to 9:10) and 71% of admissions with unnecessary delays.

• Three significant predictors of longer Score to Door Time were established: being treated in a British centre, higher APACHE II score and increasing age.

• Score to Door Time was independent of illness severity and, when combined with qualitative feedback from centres, suggests that admission delays could be due to organisational issues, rather than patient factors.

• Our tool successfully facilitated comparison, but further studies are required to establish if the observed delays in admissions ultimately affect mortality and morbidity.

## Abbreviations

APACHE II: Acute Physiology and Chronic Health Evaluation; CCO: critical care outreach; MET: medical emergency team; MEWS-A: modified early warning score-A; MRIS: Medical Research Information Service; RRS: rapid response systems; RRT: rapid response team; SPOT: Sepsis Pathophysiological and Organisational Timing; SPSS: statistical package for the social sciences; STDT, Score To Door Time; ViEWS: VitalPAC™ early warning score.

## Competing interests

LD, JW and CPS are members of the National Critical Care Outreach Forum (NOrF). NOrF is an organisation of professionals working with Rapid Response Systems in the UK. The authors declare that they have no other competing interests.

## Authors' contributions

All authors made substantial contributions to the study design, acquisition and interpretation of data, and they revised the manuscript for important intellectual content. CPS conceived the service evaluation. KO and CPS wrote the initial draft and performed the data analysis. All authors have read and approved the final manuscript.
